# CT hepatic arterial perfusion index does not allow stratification of the degree of esophageal varices and bleeding risk in cirrhotic patients in Child–Pugh classes A and B

**DOI:** 10.1007/s00261-021-03259-6

**Published:** 2021-08-27

**Authors:** Felix Peisen, Kaspar Ekert, Michael Bitzer, Hans Bösmüller, Jan Fritz, Marius Horger

**Affiliations:** 1 Department of Diagnostic and Interventional Radiology, Eberhard Karls University, Hoppe-Seyler-Strasse 3, 72076 Tübingen, Germany; 2 Department of Gastroenterology, Gastrointestinal Oncology, Hepatology and Infectious Diseases, Eberhard Karls University, Otfried-Müller-Str. 10, 72076 Tübingen, Germany; 3 Department of Pathology and Neuropathology, University Hospital Tübingen and Eberhard Karls University Tübingen, Liebermeisterstraße 8, 72076 Tübingen, Germany; 4 Department of Radiology, New York University Grossman School of Medicine, 660 1st Ave, New York, NY USA

**Keywords:** Esophageal varices, Portal hypertension, Perfusion index, Retrospective studies, X-ray computed tomography

## Abstract

**Purpose:**

To evaluate if the hepatic arterial perfusion index (HPI) in liver parenchyma of cirrhotic patients can serve as a surrogate parameter for stratifying the degree of esophageal varices and related bleeding risks.

**Methods:**

CT image data of sixty-six patients (59 men; mean age 68 years ± 10 years) with liver cirrhosis (Child–Pugh class A (35/66, 53%), B (25/66, 38%), and C (6/66, 9%) who underwent perfusion CT (PCT) for hepatocellular carcinoma (HCC) screening between April 2010 and January 2019 were retrospectively identified. HPI, a parameter calculated by a commercially available CT liver perfusion analysis software that is based on the double maximum slope model, using time attenuation curve to determine perfusion, was correlated with the degree of esophageal varices diagnosed at endoscopy and the number of bleeding events.

**Results:**

Eta correlation coefficient for HPI/presence of esophageal varices was very weak (0.083). Spearman-Rho for HPI/grading of esophageal varices was very weak (0.037 (*p* = 0.804)). Kendall-Tau-b for HPI/grading of esophageal varices was very weak (0.027 (*p* = 0.807)). ANOVA and Bonferroni post-hoc-tests showed no significant difference of HPI between different grades of esophageal varices (*F* (3, 62) = 1.676, *p* = 0.186). Eta correlation coefficient for HPI/bleeding event was very weak (0.126).

**Conclusion:**

The stratification of the degree of esophageal varices and the related bleeding risk by correlation with the HPI as a surrogate parameter for portal venous hypertension was not possible for patients with liver cirrhosis in Child–Pugh class A and B.

**Graphic abstract:**

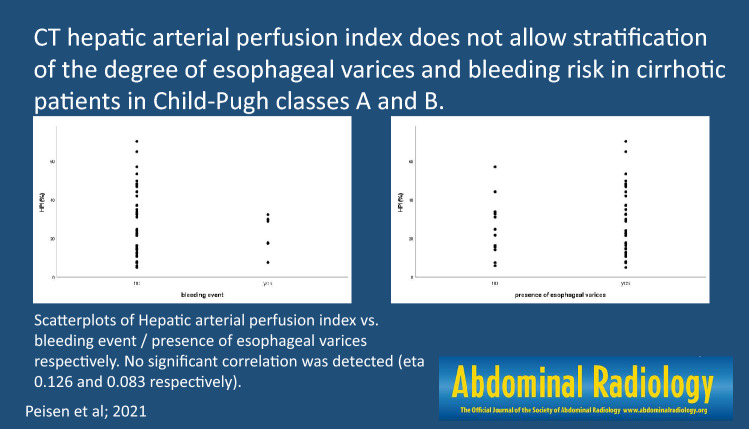

## Introduction

Patients with cirrhotic liver disease may develop portosystemic collateral vessels due to increased resistance within the portal venous system. Esophageal variceal bleeding is a potentially life-threatening complication with high mortality rates demanding emergency treatment [1]. In addition to other prognostic risk factors such as Child–Pugh score and MELD score, the presence of HCC is an independent risk factor for re-bleeding [2]. Prophylactic endoscopic monitoring and treatment of varices are often carried out to reduce variceal bleeding and improve survival [3]. However, an endoscopic maintenance program may not be feasible in every patient, especially in those with advanced HCC, due to their poor health status [2]. Therefore, Amtirano et al. promoted the identification of high-risk patients [2].

According to an older prognostic score, including Child–Pugh score, red wale markings at endoscopy and varix size can be used to predict the probability of variceal bleeding [4]. CT esophagography allows grading esophageal varices and differentiation between low- and high-risk varices and may have better patient acceptance than endoscopy [5]. Quantitative imaging parameters on abdominal CT, including intraluminal protrusion of gastric varices, gastric varix size, and larger spleen and liver volumes, were proposed as predictors of portal venous intervention [6]. However, this method requires manual caliper measurement of varices and depends on differences in the millimeter range, rendering this method prone to errors.

At our institution, cirrhotic patients regularly undergo perfusion computed tomography (PCT) for hepatocellular carcinoma (HCC) screening, in contrast to many other institutions, where ultrasound serves as the only baseline screening imaging modality for HCC screening. Despite the added costs and radiation dosage, PCT for HCC imaging provides an increased sensitivity and specificity compared to ultrasound and adds excellent anatomical resolution and reliable quantitative perfusion data [7, 8]. PCT serves as the baseline and follow-up modality when HCC is present. PCT is a technique that allows for quantification of the perfusion in tumor and liver tissue in absolute values by measuring flow and concentration of iodinated contrast medium during a period within blood vessels and tissue, generating time density curves. PCT can separately calculate hepatic arterial and portal venous blood flow to the liver and liver tumors based on input functions obtained by ROIs (region of interest) set in the spleen and the portal vein. The spleen serves as a substitute for direct hepatic arterial measurement. The hepatic perfusion index (HPI) expresses the degree of arterial supply [9]. Former publications successfully demonstrated that quantitative data from PCT (portal-venous perfusion and HPI) can be used to differentiate between liver fibrosis (F3) and liver cirrhosis (F5/F6) [10]. Other authors showed that perfusion-based entire HCC-tumor characterization was feasible using PCT when patients with venous thrombosis and TIPS were excluded [9]. Therefore, it would have been conclusive that the HPI should also correlate with the degree of esophageal varices and bleeding events, as the extent of varices is directly connected to portal hypertension [11]. A significant correlation could potentially serve as a basis for a CT-based screening method in patients that routinely undergo PCT scans.

Therefore, the purpose of our study was to evaluate if the HPI in liver parenchyma of cirrhotic patients can serve as a surrogate parameter for stratifying the degree of esophageal varices and related bleeding risks.

## Material and methods

### Ethics

This study protocol was performed according to the ethics guidelines of the Declaration of Helsinki (1975) and was approved by the local ethics committee. Due to the retrospective study design, written informed consent was waived.

### Study population

A total of 384 patients with liver cirrhosis who underwent PCT between April 2010 and January 2019 for hepatocellular carcinoma screening were identified from a local database.

**Fig. 1:**
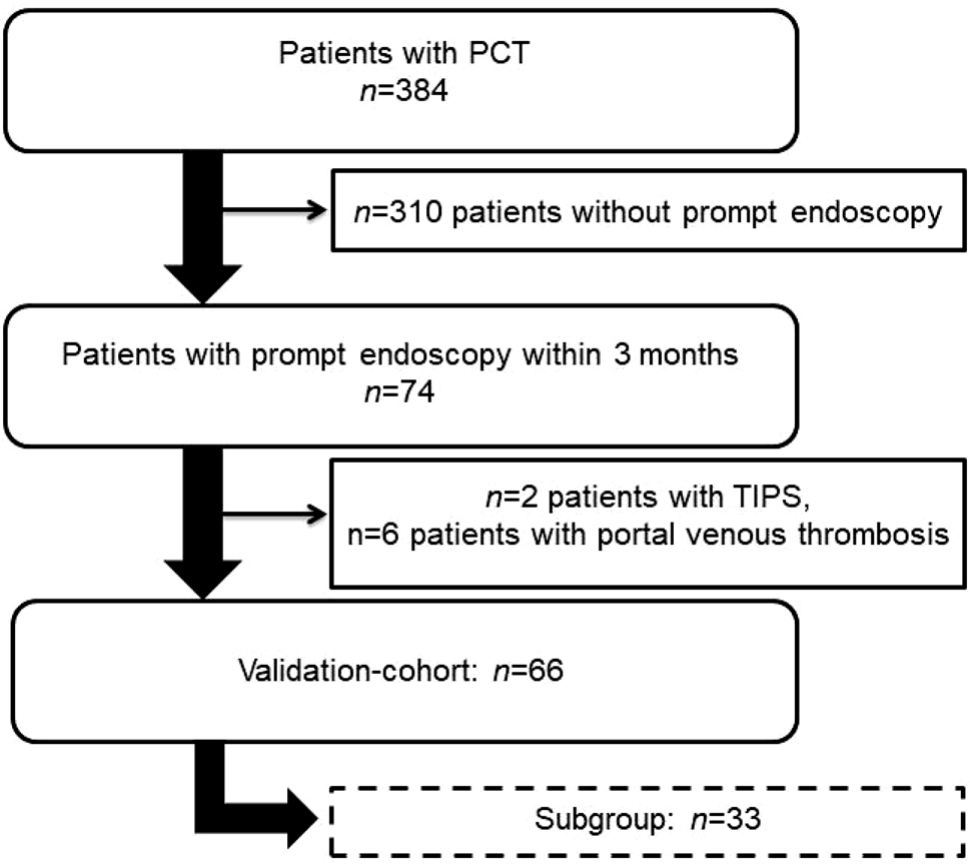
Patient selection. *PCT* perfusion computed tomography, *TIPS* transjugular intrahepatic portosystemic shunt

Inclusion criteria for this study were liver cirrhosis (diagnosed by needle biopsy), a PCT exam, and endoscopy within three months of PCT. Exclusion criteria were diffusely infiltrating HCC due to difficulties in differentiating diffusely infiltrating HCC from normal liver parenchyma, transjugular intrahepatic portosystemic shunt (TIPS), and portal venous thrombosis (see Fig. 1). Sixty-six patients (59 men; mean age 68 ± 10 years) were included in the final cohort. The underlying chronic liver diseases were HBV-infection (4/66, 6%), HCV-infection (21/66, 32%), and chronic alcohol use (32/66, 48%). In 9/66 (14%) patients, the underlying disease was unknown. 35/66 (53%) patients were classified as Child–Pugh A, 25/66 (38%) as Child–Pugh B, and 6/66 (9%) as Child–Pugh C. Table 1 summarizes the demographics. 33/66 (50%) patients with a history of variceal bleeding and prior endoscopic treatment were excluded from a subgroup analysis due to lower bleeding risks.

**Table 1:** patients characteristics

	Count	%
Total	66	100
Gender		
Male	59	89
Female	7	11
Age (years)	68(10)*	
Child–Pugh class		
A	35	53
B	25	38
C	6	9
Etiology of cirrhosis		
HBV	4	6
HCV	21	32
Alcohol abuse	32	48
n.a	9	14
Endoscopic grading of esophageal		
Varices according to Paquets		
No varices	16	24
I	25	38
II	20	30
III	5	8
Patients with HCC	45	68
BCLC Stage 0	14	21
BCLC Stage A	30	45
BCLC Stage B	1	1
BCLC Stage C	0	0
BCLC Stage D	0	0
HCC number	1.3 (0.6)*	
HCC size (mm)	16 (9)*	
Portal vein thrombosis	0	0

*BCLC* Barcelona Clinic Liver Cancer, *HBV* hepatitis B virus, *HCC* hepatocellular carcinoma, *HCV* hepatitis C virus

*Mean (SD)

### PCT imaging protocol

All examinations were performed on a 128-slice CT scanner (Somatom Definition AS + or FLASH, Siemens Healthcare, Forchheim, Germany). The CT protocol consisted of a non-enhanced abdominal low-dose CT, which was obtained to localize the portal vein. Subsequently, a fixed scan range of 6.9–9.7 cm *z*-axis coverage was planned over the involved liver, followed by a volume perfusion CT (PCT) using adaptive spiral scanning technique. The acquisition parameters are given in Table 2. To assess the optimal delay time for contrast agent administration, a test bolus with 6 mL of an iodine contrast agent was used to time the perfusion appropriately. Hence, there were only 1–2 non-enhanced series in the protocol. The mean radiation exposure for liver perfusion measurements was 7 mSv (CTDIvol: 60.6 mGy [SD: 1.73 mGy]; DLP: 1226.65 mGycm [SD: 404.04 mGycm]). During the acquisition of the perfusion data, the patients were asked to breath shallowly. 50 mL Ultravist 370 (Bayer Vital Leverkusen, Germany) was injected at a flow rate of 5 mL/s in an antecubital vein followed by a saline flush of 50 mL NaCl at 5 mL/s. The contrast medium was administered with a dual-head pump injector (Stellant, Medtron, Saarbruecken, Germany). From the PCT raw data, one set of axial images with a slice thickness of 3 mm was reconstructed for perfusion analysis without overlap, using a smooth tissue convolution kernel (B10f). All images were transferred to an external workstation (Multi-Modality Workplace, Siemens Healthcare) for analysis.

**Table 2:** CT acquisition parameters

Low dose CT	
Radiation	40 mAs
Current	100 kV
Slice thickness	5.0 mm
Collimation	128 mm × 0.6 mm
Tube rotation time	0.5 s
Pitch	0.6
PCT	
Radiation	100 mAs/120 mAs (patients > 70 kg)
Current	80 kV
Collimation	64 mm × 0.6 mm
Fixed scan range	6.9–9.7 cm *z *axis
Coverages	26
Acquisition time	40 s

*CT* computed tomography, *PCT* perfusion computed tomography

### Image analysis and quantitative perfusion assessment with PCT

Image analysis was performed by a senior radiologist with more than 30 years of expertise in gastrointestinal and hepatic imaging. All data sets were transferred to a dedicated workstation (SyngoMMWP, VE 36A, Siemens Healthcare), and quantitative data evaluation was performed with commercial software (*Syngo.* VolumePerfusion CT Body [Siemens Healthcare]). CT liver perfusion analysis software is based on the double maximum slope model, using time attenuation curve (TAC) to determine perfusion. Automated motion correction and noise reduction of the datasets were applied using an integrated motion correction algorithm with non-rigid deformable registration for anatomic alignment. 4D Noise Reduction is a frequency-dependent filter applied on the dynamic data: high spatial frequencies which do not contain relevant perfusion information are averaged to improve the Signal to Noise Ratio, while low spatial frequencies containing the dynamic perfusion information are untouched to keep the maximal perfusion information. Regions of interest were placed in the abdominal aorta for measuring the arterial input function. Additional ROIs were set in the portal vein and spleen for separate calculation of arterial and portal-venous blood supply contributions to the liver. According to the description of the vendor, calculation of arterial liver perfusion (ALP) and portal venous perfusion (PVP) was done using the time of peak splenic enhancement as a separation point of arterial and portal-venous phase by drawing regions of interest in the portal vein and the spleen, respectively. The maximum slope model was applied twice on the hepatic time attenuation curve. The underlying assumption was that the arrival time of arterial blood in the spleen and liver are similar. ALP was determined by dividing the maximum slope of the hepatic time attenuation curve before the peak spleen enhancement (arterial-dominant phase) by the peak aortic enhancement. PVP was calculated by dividing the maximum slope of the hepatic time attenuation curve after the peak splenic enhancement (portal-dominant phase) by the peak portal vein enhancement.

**Fig. 2:**
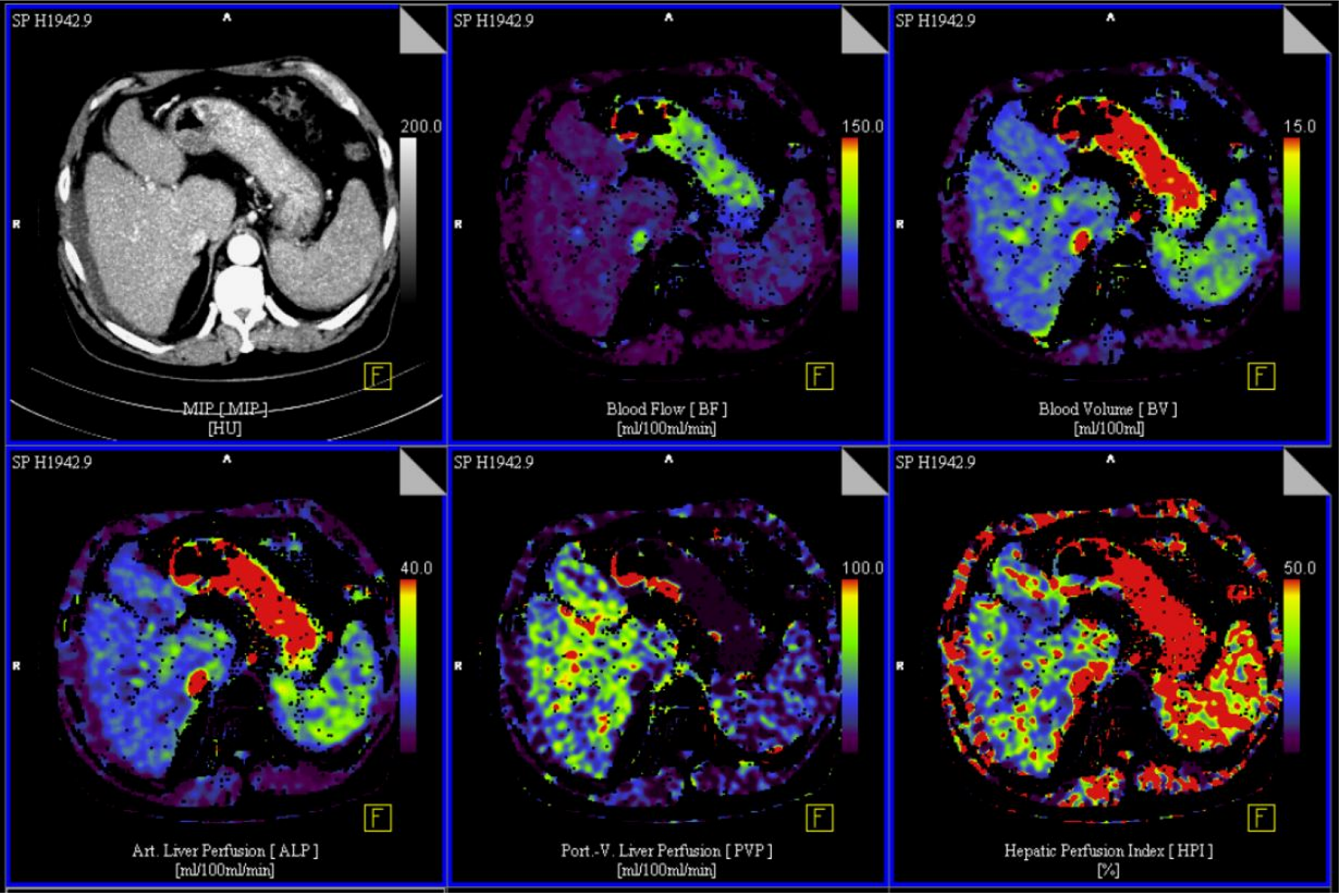
Exemplary axial slides of a perfusion computed tomography of non-tumor liver parenchyma and calculated color maps. Top left: maximum intensity protection. Top center: Blood Flow (BF). Top right: Blood Volume (BV). Bottom left: Arterial liver perfusion (ALP). Bottom center: Portal venous liver perfusion (PVP). Bottom right: Hepatic arterial perfusion index (HPI). In general, red and yellow colors indicate high values, green colors medium values and blue and violet colors low values

ALP was defined as maximum slope (arterial phase)/maximum aorta enhancement; PVP was defined as maximum slope (portal-venous phase) / maximum portal-vein enhancement; HPI was defined as ALP/(ALP + PVP) × 100%. ALP- and PVP-values were given in mL per 100 mL of tissue per minute. Blood Volume was indicated as mL per 100 mL of tissue, and HPI is indicated in percent. See Fig. 2 for an image example of a PCT of non-tumor liver parenchyma.

### Endoscopy

**Fig. 3:**
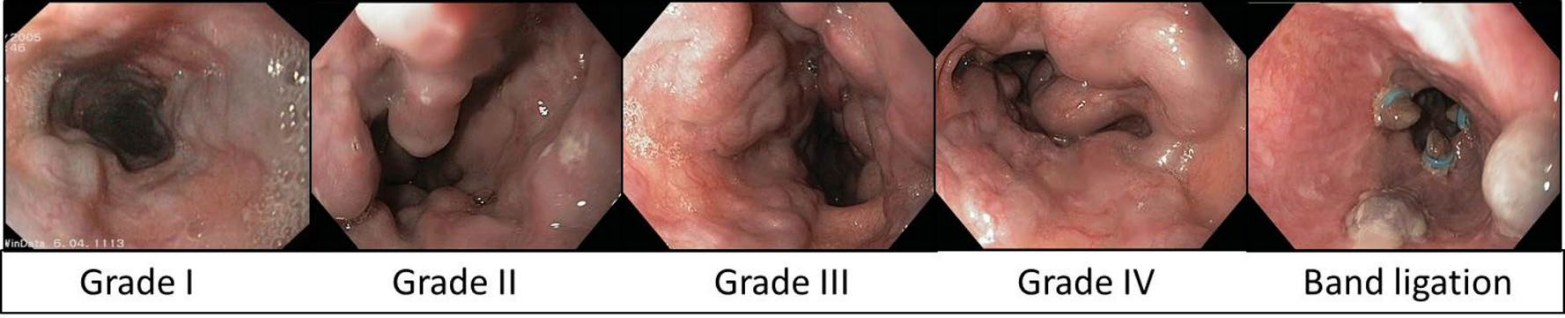
Endoscopic grading of esophageal varices according to Paquets (I: Varices surpass the level of mucous membrane, however disappear after insufflation of air, II: Varices that extend into the esophageal lumen less than 1/3 of esophageal diameter, III: Varices that extend into the esophageal lumen less than 1/2 of esophageal diameter and/or “cherry red spots”, IV: Varices that fill the esophageal lumen and often stretch into the upper third part of the esophagus, additionally “whale sign”). Images by courtesy of Michael Oelckers, Hamburg, Germany; endoskopiebilder.de/endoskopie-bilder)

Patients underwent endoscopy by a board-certified gastroenterologist with 9 years of expertise at the in-house endoscopy ward (Device: Pentax EG 2990 i, AD 9,8 mm, ID 2,8 mm) within three months of PCT. Our interdisciplinary in-house endoscopy ward is a nationally certified center with over 9000 endoscopies carried out per year. Endoscopy was performed with sedation. The presence of esophageal varices was visually graded according to the Paquets criteria (0: Absence of esophageal varices, I: Varices surpass the level of mucous membrane, however disappear after insufflation of air, II: Varices that extend into the esophageal lumen less than 1/3 of esophageal diameter, III: Varices that extend into the esophageal lumen less than 1/2 of esophageal diameter and/or “cherry red spots”, IV: Varices that fill the esophageal lumen and often stretch into the upper third part of the esophagus, additionally “whale sign”)[12]. Varices were treated with band ligation (Fig. 3).

### Statistical analysis

Statistical analyses were performed with SPSS 25.0 (IBM SPSS). Sample size and power calculation were based on the two-tailed test [13]. The aim of the study was to determine the correlation between variables derived from PCT (HPI, PVP and splenic blood flow) and clinical endpoints (bleeding event, variceal band ligation, presence of esophageal varices, Child–Pugh score, and grading of the esophageal varices). Our statistical aims included an alpha error of less than 5% with at least 80% power to detect correlation with a correlations coefficient of at least 0.5, which would be a strong correlation according to Cohen [14]. Therefore, the minimum required sample size for this study was 46. Moderate correlation (> 0.3) would have required minimum sample size of 84 [15]. Categorical data are reported as counts and percentages. Continuous data are reported as mean and standard deviation if not marked otherwise. Eta correlation coefficient was used to check for significant correlations of variables with a metric/nominal scale level (HPI/bleeding event; HPI/variceal band ligation; HPI/presence of esophageal varices) [16]. Spearman-Rho and Kendall-Tau-b were used to assess significant correlations of metric/ordinal scale variables (HPI/Child–Pugh score; HPI/grading of the esophageal varices). ANOVA and Bonferroni post-hoc-tests were used to assess significant differences of HPI between different grades of esophageal varices. The significant correlation between HPI and esophageal varices in the soubgroup analysis was further investigated with the Mann–Whitney-*U*-Test to assess significant differences in the median of HPI between patients with and patients without esophageal varices on endoscopy. A difference with a *p *value of less than 0.05 was considered significant.

## Results

### Endoscopy

8/66 (12%) patients showed bleeding during endoscopy. 12/66 (18%) patients underwent endoscopic variceal band ligation. Grading of the esophageal varices according to Paquets was as following: grade 0 (16/66, 24%), grade 1 (25/66, 38%), grade 2 (20/66, 30%), grade 3 (5/66, 8%). No patient requried a surgical intervention due to bleeding episodes during endoscopy.

### Correlation

**Fig. 4:**
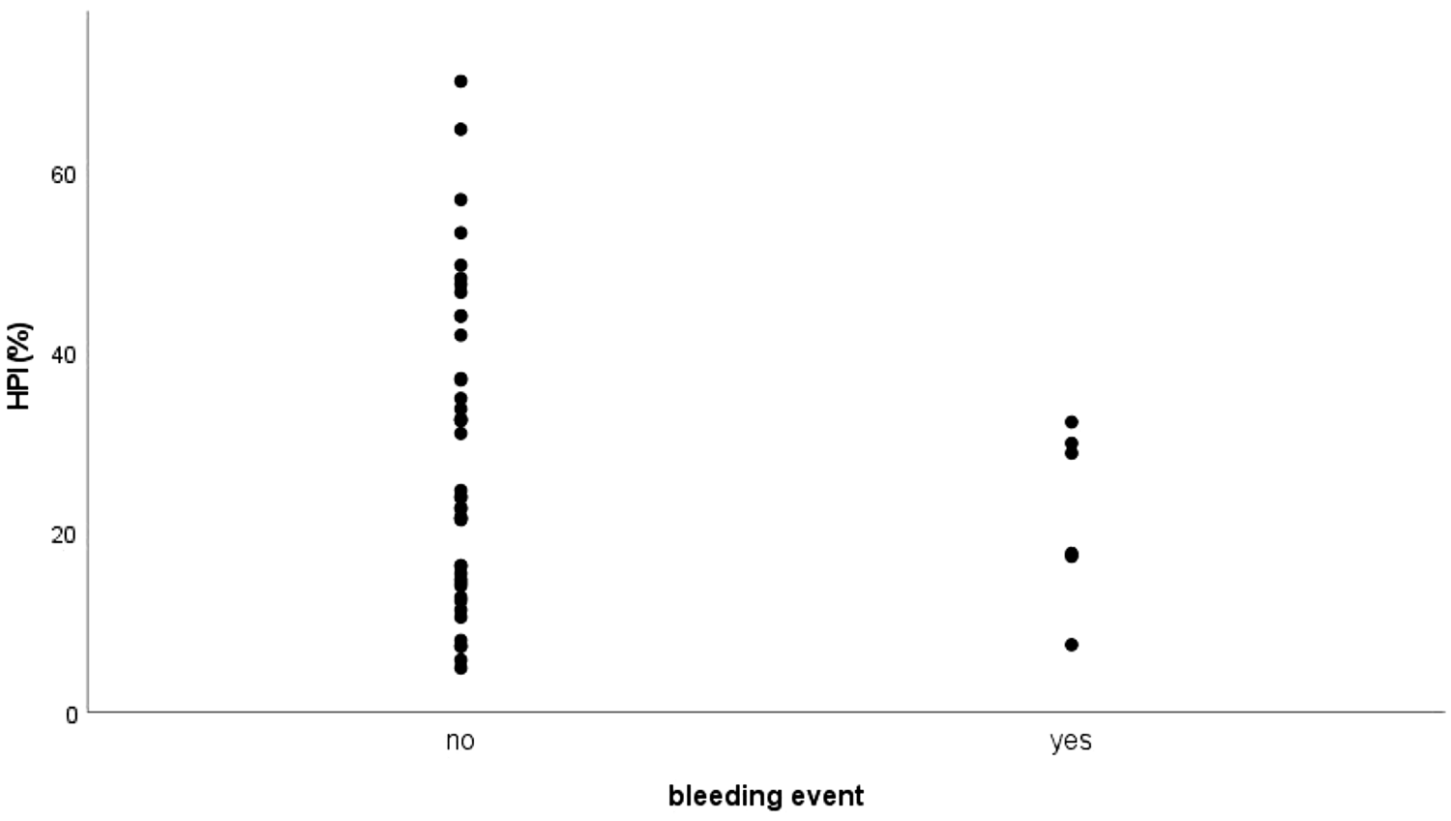
Scatterplot: Hepatic arterial perfusion index (HPI (%))/bleeding event

Correlation of HPI and bleeding event with Eta correlation coefficient was 0.126, indicating a very weak correlation (Fig. 4).

Correlation of HPI and variceal band ligation with Eta correlation coefficient was 0.232, indicating a weak correlation.

**Fig. 5:**
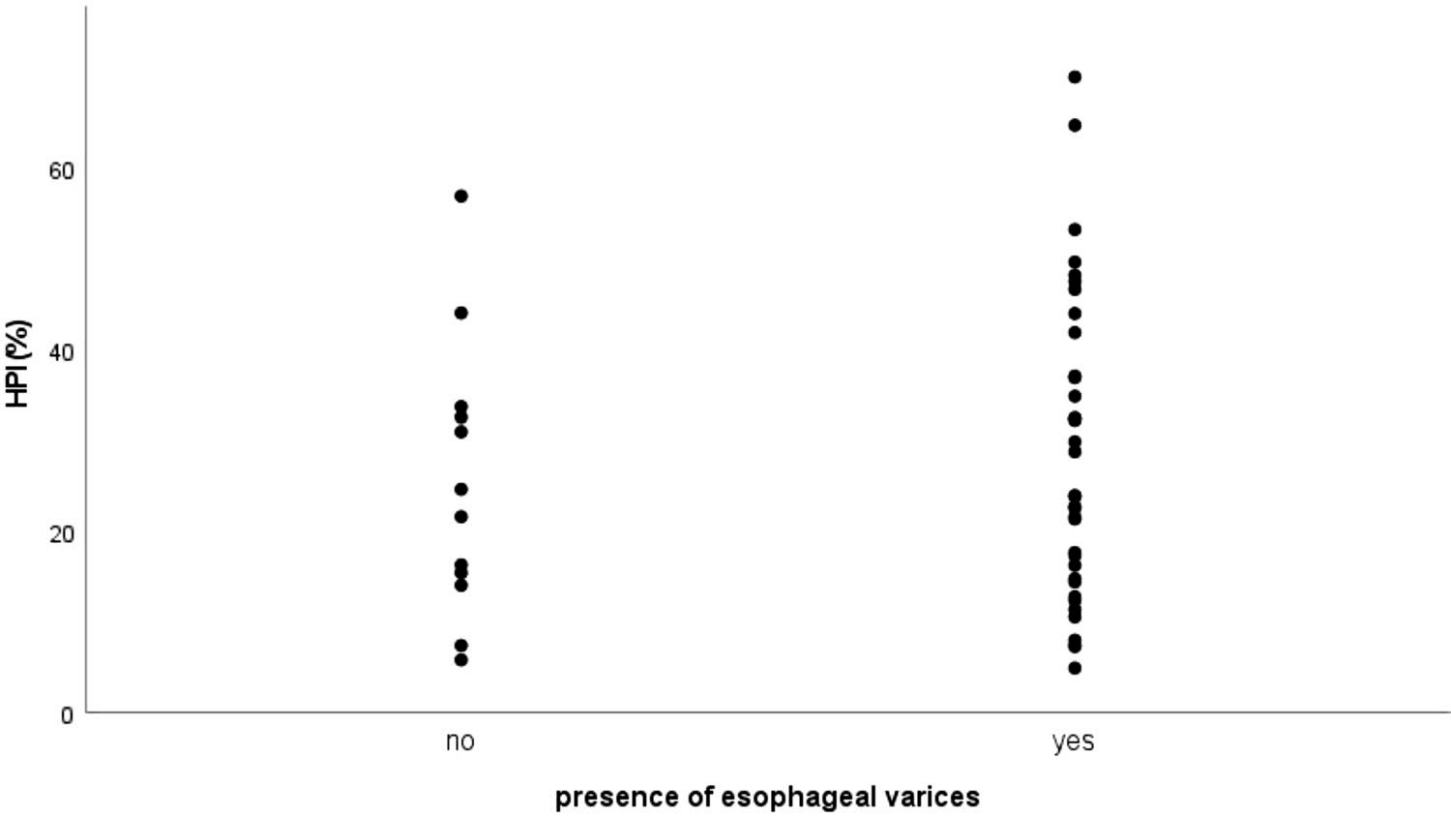
Scatter plot: Hepatic arterial perfusion index (HPI (%))/presence of esophageal varices

Correlation HPI and presence of esophageal varices with Eta correlation coefficient was 0.083, indicating a very weak correlation (Fig. 5).

Correlation of HPI and Child–Pugh score with Spearman-Rho was 0.100 (*p* = 0.544) and with Kendall-Tau-b 0.075 (*p* = 0.564), respectively. Both parameters indicated a non-significant and only very weak correlation.

**Fig. 6:**
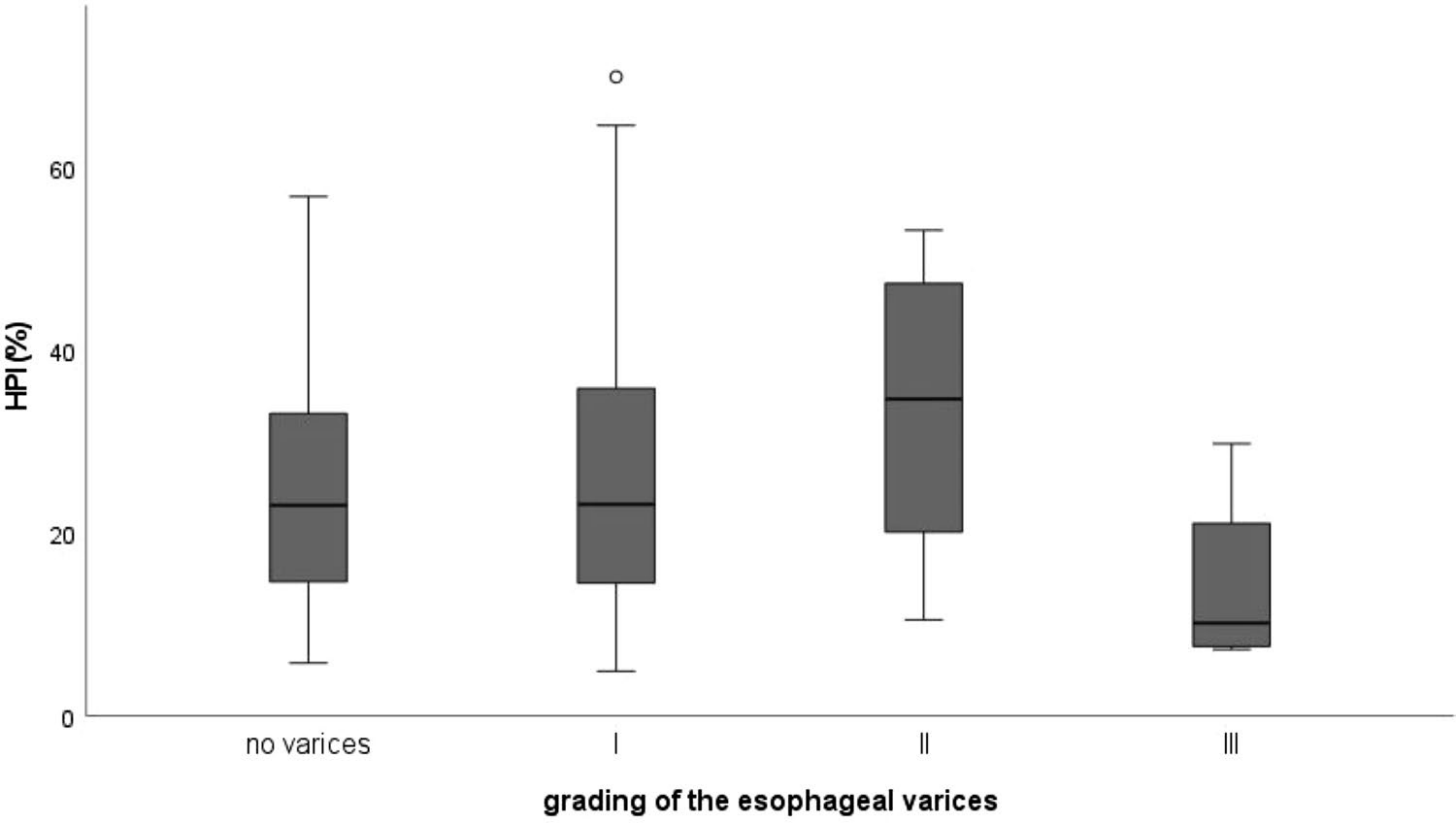
Boxplot: Hepatic arterial perfusion index (HPI (%)) for different grades of esophageal varices according to Paquets

Correlation of HPI and grading of the esophageal varices with Spearman-Rho was 0.037 (*p* = 0.804) and Kendall-Tau-b 0.027 (*p* = 0.807), respectively. Both parameters indicated a non-significant and only very weak correlation. ANOVA and Bonferroni post hoc-tests showed no significant difference of HPI between different grades of esophageal varices (*F* (3, 62) = 1.676, *p* = 0.186) (Fig. 6).

**Fig. 7:**
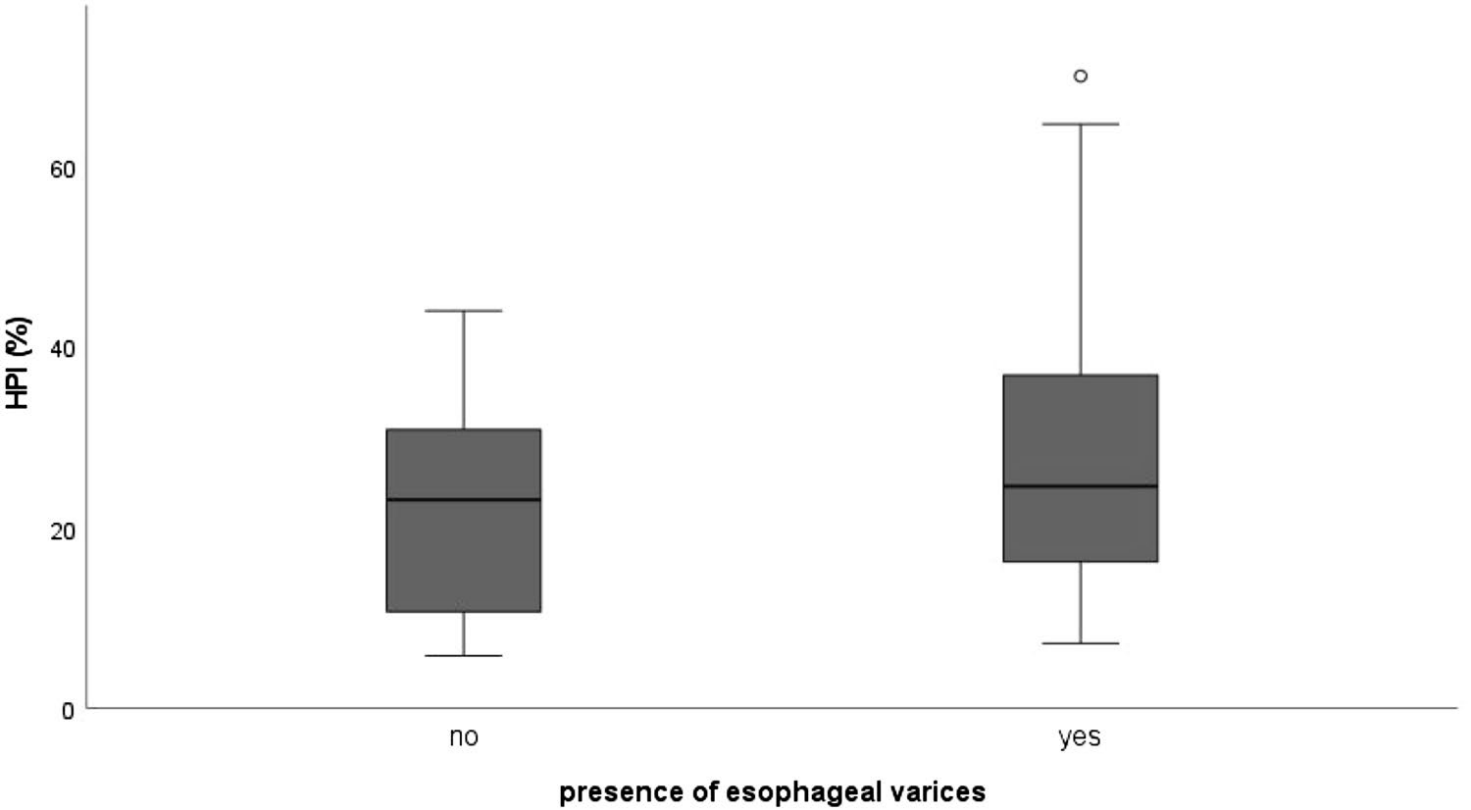
Subgroup Analysis Boxplot: Hepatic arterial perfusion index (HPI (%)) for presence of esophageal varices

As patients with a prior history of bleeding esophageal varices and/or variceal band ligation might bias the correlation analysis [17], we performed a subgroup analysis (33/66, 50%), including only patients with no prior bleeding esophageal varices and/or variceal band ligation. Table 3 provides detailed results. Eta coefficient for correlation of HPI and presence of esophageal varices was 0.384. The other coefficients showed only weak correlations of the investigated parameters. Further analysis of the correlation between HPI and presence of esophageal varices with the Mann–Whitney-*U*-Test, however, showed no significant difference of HPI between patients with (median 24.59) and patients without (median 23.08) presence of esophageal varices in endoscopy (*U* = 79.5, *p* = 0.389) (Fig. 7).

**Table 3:** correlation coefficients

HPI				
Pair	Scale level	Coefficient	Result	*p*
HPI/bleeding event	metric/nominal	Eta	0.126	
HPI/variceal band ligation	metric/nominal	Eta	0.232	
HPI/presence of esophageal varices	metric/nominal	Eta	0.083	
HPI/Child–Pugh score	metric/ordinal	Spearman-Rho	0.544	0.544
HPI/Child–Pugh score	metric/ordinal	Kendall-Tau-b	0.075	0.564
HPI/grading of the esophageal varices	metric/ordinal	Spearman-Rho	0.037	0.804
HPI/grading of the esophageal varices	metric/ordinal	Kendall-Tau-b	0.027	0.807

HPI subgroup				
Pair	Scale level	Coefficient	Result	*p*
HPI/bleeding event	metric/nominal	Eta	0.131	
HPI/variceal band ligation	metric/nominal	Eta	0.209	
HPI/presence of esophageal varices	metric/nominal	Eta	0.384	
HPI/Child–Pugh score	metric/ordinal	Spearman-Rho	0.146	0.418
HPI/Child–Pugh score	metric/ordinal	Kendall-Tau-b	0.119	0.387
HPI/grading of the esophageal varices	metric/ordinal	Spearman-Rho	0.228	0.203
HPI/grading of the esophageal varices	metric/ordinal	Kendall-Tau-b	0.177	0.194

PVP				
Pair	Scale level	Coefficient	Result	*p*
PVP/bleeding event	metric/nominal	Eta	0.031	
PVP/variceal band ligation	metric/nominal	Eta	0.015	
PVP/presence of esophageal varices	metric/nominal	Eta	0.099	
PVP/Child–Pugh score	metric/ordinal	Spearman-Rho	− 0.124	0.397
PVP/Child–Pugh score	metric/ordinal	Kendall-Tau-b	− 0.095	0.411
PVP/grading of the esophageal varices	metric/ordinal	Spearman-Rho	0.006	0.964
PVP/grading of the esophageal varices	metric/ordinal	Kendall-Tau-b	0.012	0.907

PVP subgroup				
Pair	Scale level	Coefficient	Result	*p*
PVP/bleeding event	metric/nominal	Eta	0.106	
PVP/variceal band ligation	metric/nominal	Eta	0.125	
PVP/presence of esophageal varices	metric/nominal	Eta	0.273	
PVP/Child–Pugh score	metric/ordinal	Spearman-Rho	0.027	0.883
PVP/Child–Pugh score	metric/ordinal	Kendall-Tau-b	0.032	0.820
PVP/grading of the esophageal varices	metric/ordinal	Spearman-Rho	− 0.265	0.142
PVP/grading of the esophageal varices	metric/ordinal	Kendall-Tau-b	− 0.194	0.163

BF spleen				
Pair	Scale level	Coefficient	Result	*p*
BF/bleeding event	metric/nominal	Eta	0.119	
BF/variceal band ligation	metric/nominal	Eta	0.092	
BF/presence of esophageal varices	metric/nominal	Eta	0.146	
BF/Child–Pugh score	metric/ordinal	Spearman-Rho	− 0.193	0.179
BF/Child–Pugh score	metric/ordinal	Kendall-Tau-b	− 0.148	0.194
BF/grading of the esophageal varices	metric/ordinal	Spearman-Rho	− 0.211	0.098
BF/grading of the esophageal varices	metric/ordinal	Kendall-Tau-b	− 0.159	0.101

BF spleen subgroup				
Pair	Scale level	Coefficient	Result	*p*
BF/bleeding event	metric/nominal	Eta	0.038	
BF/variceal band ligation	metric/nominal	Eta	0.078	
BF/presence of esophageal varices	metric/nominal	Eta	0.066	
BF/Child–Pugh score	metric/ordinal	Spearman-Rho	− 0.226	0.221
BF/Child–Pugh score	metric/ordinal	Kendall-Tau-b	− 0.180	0.202
BF/grading of the esophageal varices	metric/ordinal	Spearman-Rho	− 0.157	0.398
BF/grading of the esophageal varices	metric/ordinal	Kendall-Tau-b	− 0.130	0.357

*BF* blood flow, *HPI* hepatic perfusion index, *PVP* portal venous perfusion

HPI is a calculated parameter consisting of the ratio of portal venous perfusion and arterial liver perfusion. As HPI itself did not correlate with the investigated parameters, further investigation of two more robust parameters (PVP and perfusion of the spleen) was carried out. The calculation of correlation parameters of PVP and perfusion of the spleen with bleeding events, variceal band ligation, presence of esophageal varices, Child–Pugh score, and grading of the esophageal varices did only show weak and non-significant results. This also applied to the subgroup analysis, which was carried out in accordance with HPI correlations. Detailed values are given in Table 3.

## Discussion

Most patients with chronic liver diseases develop different degrees of liver fibrosis and portal hypertension, the latter being accompanied by the formation of portosystemic collateral vessels like esophageal varices [18]. Varices are a potentially life-threatening source of bleedings and are regularly monitored and treated via endoscopy [19]. Endoscopy itself, however, poses several risks, not only in patients with cirrhosis [20, 21]. A noninvasive risk-stratification for potential bleeding events and the degree of esophageal varices would thus be desirable.

Several laboratory risk factors for esophageal bleeding in cirrhotic patients and predictors for short-term mortality have been identified. Amitrano et al. promoted the MELD score as an indicator for short-term mortality among cirrhotic patients at their first episode of bleeding from esophageal varices [2]. Sanyal et al. connected the risk of developing varices to decreased platelet counts, increased bilirubin, and increased INR [22].

Other studies have identified and evaluated CT findings (size of esophageal and gastric varices, protrusion of gastric varices, hepatomegaly, splenomegaly, and ascites), most of them relying on manual measurements [6, 23, 24].

However, a more objective noninvasive stratification with radiographic methods such as perfusion computed tomography based on accurate quantification of the dual blood supply to the liver has not been investigated yet. As several patients at risk for HCC regularly undergo PCT at few institutions [8, 25], we hypothesized that the hepatic perfusion index could serve as a surrogate parameter for the risk of bleeding in patients with portal hypertension and esophageal varices and might correlate with the extent of esophageal varices.

However, our study data show that this hypothesis must be rejected. Accordingly, the eta correlation coefficient showed neither a positive nor a negative correlation of HPI and bleeding events or the need for variceal band ligation, respectively. Also, the presence of esophageal varices itself did not correlate with the HPI. Grading of the esophageal varices according to Paquets and the degree of liver fibrosis according to Child–Pugh did not show a correlation with the HPI. Analysis of variance revealed that the HPI could not estimate the extent of the esophageal varices according to Paquets.

As patients with a prior history of bleeding esophageal varices and/or variceal band ligation might have biased the correlation analysis [17], we performed a subgroup analysis (*n* = 33), including only patients with no prior history of bleeding esophageal varices and variceal band ligation. However, even in this subgroup analysis, there was no significant correlation between the investigated parameters and the HPI.

As mentioned in the introduction, we hypothesized that the HPI would correlate with the degree of esophageal varices and bleeding events, as the extent of varices is directly connected to portal hypertension [11]. However, this was not the case in our patient population. There are several potential explanations. The hemodynamic principles of portal hypertension, including increased intrahepatic resistance and hyperdynamic circulation, are complex with subsequent collateral circulation and varices. Feldman, Friedman, and Brandt described the underlying pathophysiological processes in detail [26].

Portal hypertension mainly results from changes in portal resistance in combination with changes in portal inflow. Increased portal resistance is, in principle, a result of mechanical factors that reduce vessel diameter. Hepatic vasoconstriction and resistance to vasodilatory stimuli (such as NO) also increase the portal resistance. Hyperdynamic circulation is an additional factor for portal hypertension. The total blood volume draining into the portal circulation, not necessarily the portal vein, is caused particularly through vasodilatation in the splanchnic bed.

Under these circumstances, the collateral circulation subsequently develops and expands in response to the increased portal pressure. Progression of portal hypertension results from the prominent obstructive resistance in the liver, resistance within the collaterals themselves, and continued increase in portal vein inflow.. Moreover, in the context of the law of Laplace, other local factors that increase variceal wall tension (transmural pressure gradient between the variceal lumen and esophageal lumen, the variceal radius, and variceal wall thickness) are also required for varices to form and bleed. However, the changes in portal pressure and local variceal factors are dynamic and influenced by several physiologic (i.e., increase in intra-abdominal pressure, meal-induced increases in portal pressure), diurnal (circadian changes in portal pressure), and pathophysiologic (acute alcohol use) factors. Therefore, portal pressure and esophageal variceal pressure may vary over time [26–29].

In the context of all these different complex mechanisms behind portal hypertension, it becomes clear that our approach quantifying the hepatic perfusion index only covers a part of the underlying causes for the development of esophageal varices and potential bleeding.

A rationale for the exclusion of patients with portal venous thrombosis was a publication that emphasized the presence of portal venous thrombosis as an independent risk factor for aggravation of esophageal varices in patients with hepatocellular carcinoma [3]. It would have, therefore, been difficult to rule out thrombosis as a possible confounder. However, this means that patients with an extremely high risk for esophageal bleeding were excluded from the cohort, and the study might therefore be underpowered.

Our study has other limitations. First, the reason for the conduction of PCT in the present cohort was to rule out HCC or exclude additional HCC manifestations before transplantation or resection. Therefore, the small present dataset was analyzed in a single-center retrospective study design. This resulted in only 66 eligible patients for inclusion. The sample size was sufficient for detecting strong correlations (> 0.5). However, the detection of significant moderate-sized correlations was not possible, which would have required a minimum of 84 patients. Furthermore, there was a disproportioned distribution of Child–Pugh class within the sample. Thus, the cohort consisted of patients with liver cirrhosis mainly in Child–Pugh class A (35/66, 53%) and B (25/66, 38%), and the main proportion of patients had only low grade or no present esophageal varices in endoscopy, which might be the reason why only 12% had bleeding events and 18% underwent variceal ligation. Unfortunately, due to the retrospective study design, it was impossible to include more patients to account for the mentioned disproportions. In summary, the sample size, especially of patients with liver cirrhosis in Child–Pugh class C (6/66, 9%), might have been too small and underpowered to detect a significant correlation of HPI and Child–Pugh class or variceal grades. Therefore, the results must be limited to patients in Child–Pugh class A and B. Furthermore, the study used only indirect data for the extent of portal hypertension (Child–Pugh score for the extent of liver cirrhosis and HPI). Additional clinical data such as Doppler sonography of the portal vein and invasive measurements of the portal venous and hepatic venous pressures using percutaneous transhepatic catheterization and venous catheterization, respectively, are missing.

In summary, our study data did not show a strong correlation between HPI and the degree of esophageal varices and variceal bleeding, potentially due to a lack of statistical power. Visual identification of the degree of esophageal varices via endoscopy and risk stratification for bleeding with MELD score, Child–Pugh score, or visual CT-parameters such as intraluminal varix protrusion, varix size as well as liver and spleen volume seems to be more robust than noninvasive parameters using PCT.

## Conclusion

The stratification of the degree of esophageal varices and the related bleeding risk by correlation with the hepatic arterial perfusion index as a surrogate parameter for portal venous hypertension was not possible for patients with liver cirrhosis in Child–Pugh class A and B.

## Abbreviations

ALP: Arterial liver perfusion
BCLC: Barcelona clinic liver cancer
BF: Blood flow
HBV: Hepatitis B virus
HCV: Hepatitis C virus
HCC: Hepatocellular carcinoma
HPI: Hepatic arterial perfusion index
MELD: Model for end-stage liver disease
PCT: Perfusion computed tomography
PVP: Portal venous perfusion
ROI: Region of interest
SD: Standard deviation
TIPS: Transjugular intrahepatic portosystemic shunt
